# The value of evidence synthesis in randomised controlled trial (RCT) design, conduct and analysis: MRC clinical trials unit (CTU) at UCL experience

**DOI:** 10.1186/1745-6215-16-S2-O4

**Published:** 2015-11-16

**Authors:** Jayne F Tierney, Sarah Burdett, Claire Vale, Larysa Rydzewska, Matthew R Sydes, Ruth E Langley, Richard S Kaplan, Mahesh KB Parmar

**Affiliations:** 1 MRC Clinical Trials Unit at UCL, London, UK

## Background

Systematic reviews of RCTs are recognised as the optimal way to resolve or confirm uncertainty about the effects of interventions. They can provide the rationale for the design of a new trial, inform the conduct of an ongoing trial, and help place trial results in the context of those from related trials. However, empirical evidence suggests that few trials use systematic reviews in these ways.

## Methods

To describe how MRC CTU has actively used evidence synthesis to inform the design, conduct and analysis of its cancer trials.

## Results

Initially, we used systematic reviews/searches to aid the interpretation and reporting of the results of MRC CTU cancer trials in light of other evidence. Subsequently, we have synthesised external evidence, to provide the rationale for new trials or protocol modifications for ongoing trials, for example, in response to new predictive biomarkers emerging during their conduct. Currently, systematic reviews are informing adaptations to our ongoing, multi-arm, multi-stage cancer trials, for example, to select the most appropriate therapies for new arms. We also plan to use prospective, retrospective and network meta-analyses of aggregate data and IPD, to tailor evidence synthesis to the specific needs of our ongoing trials, in the context of other trial evidence. We will present examples of evidence synthesis informing lung, colorectal, breast, upper GI and prostate cancer trials.

## Conclusions

Evidence synthesis has a key role to play at all stages of trials, particularly those conducted in the long term. We discuss the implications for those conducting such trials.

